# Estimating Free and Added Sugar Intakes in New Zealand

**DOI:** 10.3390/nu9121292

**Published:** 2017-11-27

**Authors:** Rachael Kibblewhite, Alice Nettleton, Rachael McLean, Jillian Haszard, Elizabeth Fleming, Devonia Kruimer, Lisa Te Morenga

**Affiliations:** Department of Human Nutrition, University of Otago, P.O. Box 56, Dunedin 9054, New Zealand; kibra436@student.otago.ac.nz (F.R.K.); netal083@student.otago.ac.nz (A.N.); Rachael.mclean@otago.ac.nz (R.M.); jill.haszard@otago.ac.nz (J.H.); liz.fleming@otago.ac.nz (E.F.); devonia.kruimer@otago.ac.nz (D.K.)

**Keywords:** dietary sugars, added sugars, free sugars, sucrose, carbohydrate, dietary intake, dietary guidelines, nutrition recommendations, population survey, New Zealand

## Abstract

The reduction of free or added sugar intake (sugars added to food and drinks as a sweetener) is almost universally recommended to reduce the risk of obesity-related diseases and dental caries. The World Health Organisation recommends intakes of free sugars of less than 10% of energy intake. However, estimating and monitoring intakes at the population level is challenging because free sugars cannot be analytically distinguished from naturally occurring sugars and most national food composition databases do not include data on free or added sugars. We developed free and added sugar estimates for the New Zealand (NZ) food composition database (FOODfiles 2010) by adapting a method developed for Australia. We reanalyzed the 24 h recall dietary data collected for 4721 adults aged 15 years and over participating in the nationally representative 2008/09 New Zealand Adult Nutrition Survey to estimate free and added sugar intakes. The median estimated intake of free and added sugars was 57 and 49 g/day respectively and 42% of adults consumed less than 10% of their energy intake from free sugars. This approach provides more direct estimates of the free and added sugar contents of New Zealand foods than previously available and will enable monitoring of adherence to free sugar intake guidelines in future.

## 1. Introduction

Intake of free or added sugars has been linked to several adverse outcomes, including weight gain [1], dental caries [2], cardiovascular disease mortality [3], type II diabetes [4], and cardiometabolic risk [5]. Restriction of free or added sugars to prevent non-communicable disease is a feature of national dietary recommendations worldwide. The World Health Organisation (WHO) recommends that individuals reduce intakes of free sugars throughout their life-course to less than 10% of total energy intake (TE) (strong recommendation) on the basis of evidence showing that free sugars contribute to weight gain and dental caries [6]. WHO also make a conditional recommendation for the reduction of free sugars intakes to below 5% TE for additional benefit. Similarly, the United States Department of Agriculture (USDA) advises limiting added sugars intakes to no more than 10% of total calorie intake to avoid excess energy intake and probable weight gain [7]. The recent inclusion of added sugars to nutrition information labels on packaged foods in the United States (US) means that consumers can now more easily check how much added sugar they are consuming [8]. However there is a need for reliable methods for measuring intakes of free or added sugars at the population level. Free and added sugars refer to sugars that are added to foods and beverages as sweeteners and include table sugar, honey, corn and other syrups, and fruit concentrates providing little in the way of nutrient value other than energy. Intrinsic sugars occur naturally in fruits, vegetables, and dairy products, and typically come bundled with additional nutrients such as dietary fibre, vitamins, and minerals [6,9]. Because free and added sugars cannot be differentiated from intrinsic sugars by laboratory-based analytical methods monitoring population intakes of free or added sugars is challenging.

Based on the WHO recommendations the New Zealand Eating and Activity Guidelines recommend limiting intakes of drinks and food with added sugars [10]. The most recent New Zealand Adult Nutrition Survey was conducted in 2008/09 (2008/09 NZANS) and reported sugar intakes based on intakes of individual mono- and disaccharides and total sugars, but not free or added sugars [11]. Being able to quantify free and added sugars is necessary to confirm that New Zealanders are indeed consuming more free sugars than ideal, to monitor changes in population free and added sugar intakes over time, to identify which population groups would benefit most from sugar reduction interventions, and to monitor the effectiveness of such interventions. 

To date there are no standardised published methods for estimating free and added sugars in foods. Few countries include free or added sugars for selected foods in their food composition tables, and when they do this information is largely derived from ingredient lists on food labels or nutrient values for total sugars in processed foods where naturally occurring sugars are assumed to be absent [12,13]. Published methods developed to estimate added sugars tend to be country-specific, are overly complicated, or provide insufficient detail to be used by other researchers [14]. Most methods involve subtracting the intrinsic sugars from the total sugar content to provide an estimate of added sugars [14,15]. This poses limitations with respect to both accuracy and repeatability, as it requires in-depth knowledge of food composition. It may also require supplementary information from food manufacturers, which is often difficult to obtain due to the frequency at which many food products are reformulated [14]. 

Therefore, the aim of the current study was to develop free and added sugar estimates for all food items and recipes in the New Zealand food composition database (FOODfiles 2010) and to estimate free and added sugar intakes in the New Zealand population based on the 2008/09 NZANS [11].

## 2. Materials and Methods 

### 2.1. Definition of Free and Added Sugars

Free sugars were defined according to the WHO definition [6]:

“Free sugars include monosaccharides and disaccharides added to foods and beverages by the manufacturer, cook or consumer, and sugars naturally present in honey, syrups, fruit juices, and fruit juice concentrates.”

[6]

Added sugars were defined according to the United States Food and Drug Administration (US FDA) definition:

“sugars that are either added during the processing of foods, or are packaged as such, and include sugars (free, mono- and disaccharides), sugars from syrups and honey, and sugars from concentrated fruit or vegetable juices that are in excess of what would be expected from the same volume of 100 percent fruit or vegetable juice of the same type. The definition excludes fruit or vegetable juice concentrated from 100 percent fruit juice that is sold to consumers (e.g., frozen 100 percent fruit juice concentrate) as well as some sugars found in fruit and vegetable juices, jellies, jams, preserves, and fruit spreads.”

[16]

Total sugars were defined as all naturally occurring and added sugars in food.

### 2.2. Development of Free and Added Sugars Database

Estimates of free and added sugar content were were added to the FOODfiles 2010 electronic data files from the New Zealand food composition database [17] by adapting the ten-step method proposed by Louie et al. (2015) for use in Australia [14]. 

The FOODfiles 2010 database contains 2779 unique food records, including both single ingredient, packaged foods, and mixed-dishes [17]. Kai-culator dietary assessment software (version 1.12 Department of Human Nutrition, University of Otago) was used to extract data for each food item listed in FOODfiles, including water, total energy, dietary fibre, polysaccharides, available carbohydrates, fructose, glucose, lactose, maltose, sucrose and total sugar per 100 g. 

**Figure 1:**
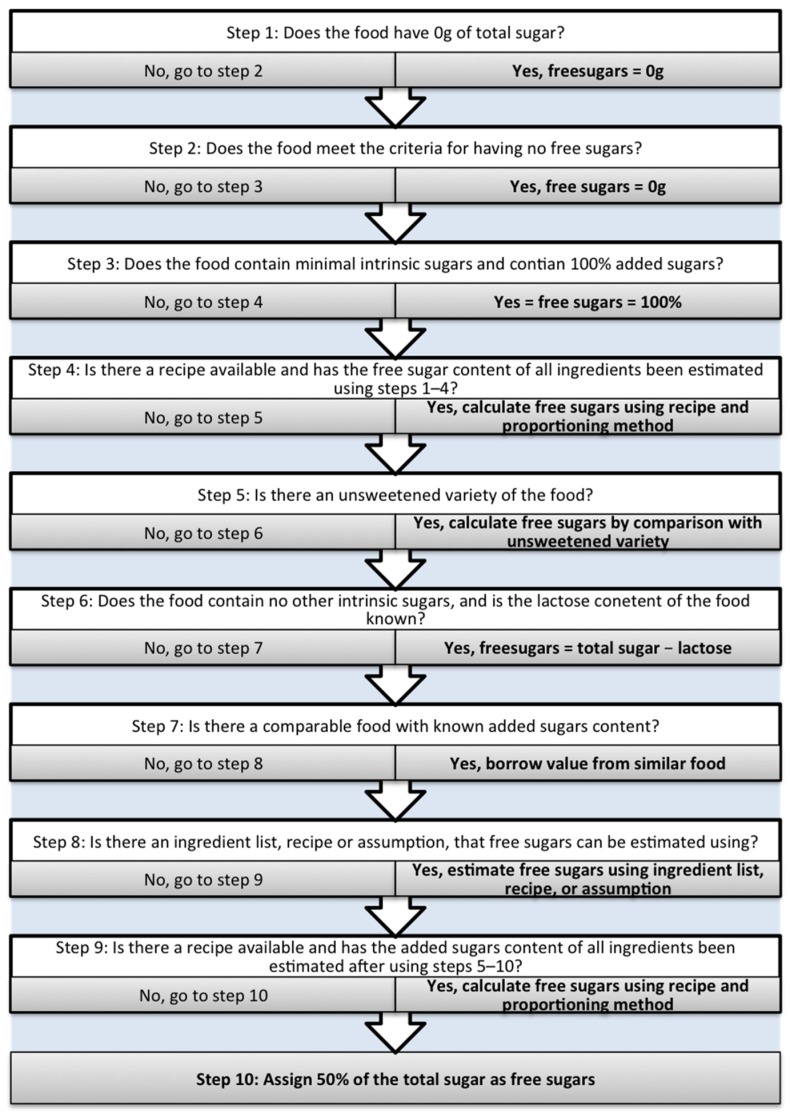
10-step method for estimating free sugars content.

Added sugars were estimated using the method described by Louie et al. 2015 [14]. Free sugars were estimated by adapting this method as described in Figure 1. The method involves six steps (1–6) using objective decisions and four steps (7–10) using subjective decisions. Modifications included changes to the types of foods categorised under steps 2 and 3, for example fruit juices, and an additional step for canned fruits in step 4. Further details are provided in the Appendix A.

Two researchers developed free and added sugar estimates for all food items in duplicate. Where discrepancies existed, decisions were adjudicated by the senior authors. For subjective decisions where a food’s ingredient list was required, researchers attempted to locate the food item in local food stores. If a product could not be found locally, then online searches were conducted to find relevant nutritional information for the product or a similar product. When multiple sources of nutritional information were available, information listed on New Zealand food product websites was prioritised. In some cases food products were no longer commercially available, thus sugar contents were estimated from similar products. 

### 2.3. Estimating Population Intakes of Free and Added Sugars

The 2008/09 NZANS was a national population-based nutrition survey with a target population of civilian individuals, living in permanent private dwellings in New Zealand, and aged 15 years and over, conducted by the Ministry of Health and University of Otago over 2008–2009. A detailed description of the survey methods can be found elsewhere [18]. To enable robust comparisons by population sub-groups, oversampling of Maori and Pacific people, and of adults in age groups 15–18 years and 71+ years, was undertaken. 

Dietary data was collected by multiple-pass 24 h diet recall (*n* = 4721), with a further 25% of participants undergoing a secondary 24 h recall to allow adjustment for intra-individual variation. Further information was collected regarding sociodemographic data, dietary habits, supplement use, and nutrition-related health. A total of 11,850 unique food descriptors were described by participants. The 24 h recall data was analysed with Kai-culator to produce a new nutrient line for each participant, including free and added sugars. 

### 2.4. Statisical Methods

All statistical analysis was undertaken on Stata/IC 15.0 (StataCorp, College Station, TX, USA). To account for intra-individual variation, nutrient intake values were adjusted using the Multiple Source Method (MSM) [19] using the subsample of repeat diet recalls that were collected. All analyses used the Stata “svy” command to account for the complex sampling design of the adult nutrition survey. Median, 25th, and 75th percentile intakes were calculated for added sugars (g), free sugars (g), total sugars (as a percentage of energy intake), sucrose (as a percentage of energy intake), added sugars (as a percentage of energy intake), and free sugars (as a percentage of energy intake). These descriptive statistics were also stratified by age and sex subpopulations. To illustrate free and added sugar intakes by gender, box plots by sex were generated. We also examined intakes by ethnicity. Where individuals identified with more that one ethnic group we assigned ethnicity by prioritisation order: 1 - Maori; 2 - Pacific; 3 - New Zealand European and Others (NZEO).

We calculated the proportion (95% confidence interval) of the population meeting the WHO [6] guidelines for free sugars and the USDA recommendations for added sugars [7], by sex and age, using two cut-offs (<10% and <5% of energy, respectively). 

## 3. Results

Overall, free, and added sugar estimates were generated for 2779 foods contained in FOODfiles 2010 [17]. Objective steps 1–6 were used to estimate free sugars for 2543 (92%) food items and added sugars for 2463 (87%) food items. Subjective steps (7–10) were used to estimate free sugars for 236 (8%) food items and added sugars for 316 (11%) food items (Table 1). Free and added sugars were estimated for 76% and 75% of food items, respectively, using the most straightforward steps (1–3).

### 3.1. Estimates of Intakes of Free Sugars and Added Sugars in New Zealand

**Figure 2:**
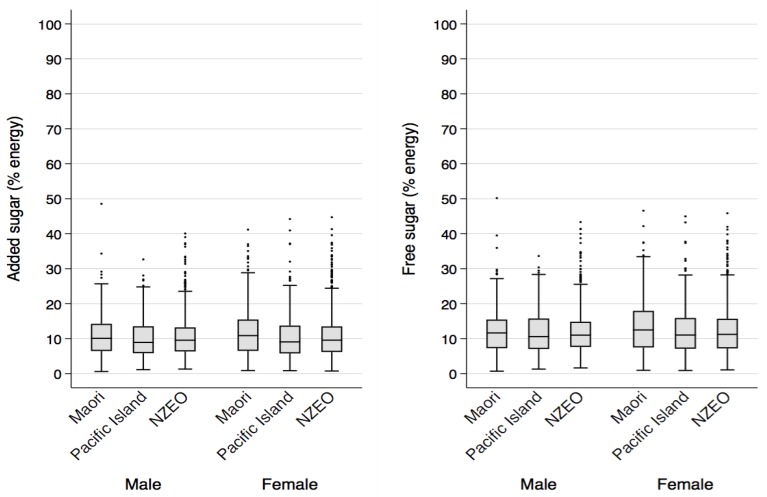
Box plots of added and free sugars intakes (as a percentage of energy intake) in New Zealand adults aged 15 years and older in 2008/09 by sex and ethnicity. The middle line is the median, the box represents the 25–75th percentiles, and the points outside the ‘whiskers’ are points that were greater than 1.5 times the interquartile range above the upper quartile. NZEO = New Zealand European and Others.

The median estimated intake of free sugars in New Zealand adults aged 15 years or older was 57.3 g per day (95% CI: 55.6, 59.1) and the median estimated intake of added sugars was 48.9 g per day (95% CI: 46.8, 51.0) (Table 2). Estimated daily intakes of free and added sugars were higher in males compared to females (males: median 66 g/day; females: median 49 g/day for free sugars, and males: 58 g/day; females: 42 g/day for added sugars). Free and added sugar intakes as a percentage of energy intake were 11% and 10%, respectively, for both men and women. Added sugar estimates were similar to estimates of sucrose intake, whereas free sugar intake estimates were slightly higher. Figure 2 presents the distribution of free and added sugar intakes (as a percentage of energy) by Maori, Pacific, and New Zealand European and Other (NZEO) adults. There were no significant differences in free and added sugar intakes by ethnicity.

### 3.2. Comparison of Reported Intakes of Free Sugars with WHO Guidelines

The WHO recommendation that intakes of free sugars should be less than 10% of daily total energy intake was met by 42% of the New Zealand adult population (Table 3). Only 27% of younger adults (15–30 years) met this recommendation. Amongst the oldest adults (aged 71+ years), 51% of the population met the recommendation for intakes of free sugars of less than 10% TE. Women were more likely to meet the recommendation than men overall, but younger women were less likely to meet the recommendation than younger men (15–30 years). A higher proportion of New Zealand adults (53%) reported consuming less than 10% of total energy from added sugars than from free sugars. Only 12% of New Zealand adults met the conditional WHO recommendation that intakes of free sugars should be less than 5% of total energy intake. 

## 4. Discussion

We adapted a ten-step systematic method for estimating added sugars in all foods listed in the Australian food composition database to estimate free and added sugar contents of all foods recorded in the New Zealand food composition database (FOODFiles). We then applied these estimates to 24 h recall data from the 2008/09 NZANS to estimate free and added sugar intakes. We found that the median intakes of added and free sugars were 49 g/day and 57 g/day, which contributed 9.6% and 11.1% of total energy intake, respectively. We estimated that only 12% of the adult population 15 years and above were meeting the WHO conditional recommendation of limiting free sugar intakes to <5% TE, and 42% (95%CI 39.7, 44.0) were meeting the WHO recommendation to limit free sugar intakes to <10% TE. Population free and added sugar intakes were higher amongst the youngest age groups (especially 15–30 years), and higher for men than women. Intakes of free and added sugars by ethnicity as a percentage of total energy intake were similar. 

This study offers an improved estimate of the current intakes of free sugars in New Zealand, providing evidence that reductions in intakes of free and added sugar would be beneficial, especially for young New Zealanders aged 15–30 years. Previously, free or added sugar intakes in New Zealand have been estimated using total sugar intake data, food comsumption data, or sucrose intakes as a surrogate measure. Total sugar and consumption data overestimate free or added sugars. In the 2008/09 NZANS the median usual daily intake of total sugar was estimated to be 120 g/day for males and 96 g/day for females [11]. Based on data reported by the Food and Agriculture Organisation, per capita daily consumption of added sugar was estimated to be 147 g/day [20]. Using sucrose as a surrogate measure of free or added sugars gave estimates of 55 g/day for males and 42 g/day for females in the 2008/09 NZANS [11]. We found that sucrose understimated free sugar intakes, and although it was a reasonable surrogate measure for added sugar intake at the total population level, it tended to overestimate added sugar intakes in older adults and underestimate intakes in younger adults. Sucrose is found in substantial quantities in processed food, as well as in fruits and vegetables, accounting for approximately 50% of total sugars in fruits such as bananas, apples, oranges, and stone fruits. Thus, sucrose will overestimate added sugar intakes in individuals or population groups for whom fruit and vegetables make an important contribution to food intake.

There is widespread consensus that excessive intakes of free sugars in processed foods and drinks are an important determinant of obesity-related non-communicable diseases. The availability of direct estimates of free and added sugars enables monitoring of population intakes and evaluation of public health strategies designed to reduce intakes. Our research indicates that sugar consumption is greater in younger populations and that sugar reduction policies targeted at this group are likely to be the most effective. Non-alcoholic sweetened beverages are the most important contributor to sugar intakes in younger New Zealand adults [11]. Therefore, a tax or levy on sugary drinks is one possible strategy that could be implemented to reduce intakes. However, critics of this approach argue that a sugary drink tax might simply lead consumers to switch to cheaper brands or to increase intake of free sugars from other food sources [21]. The availability of data on the free sugar contents of all foods listed in the FOODfiles means that we could now test the veracity of this argument. Inclusion of free sugar content on nutrition information panels is another potential strategy for reducing sugar intakes by helping consumers to make healthier food choices. Research on food labelling conducted in Australia and New Zealand has shown that many consumers intepret ‘no added sugars’ food claims to mean a product contains no sugar [22]. Our method for estimating free sugars in New Zealand makes it possible to provide consumers with better, less ambiguous information. 

The strengths of the ten-step systematic method used to develop free and added sugar estimates in this study include its simplicity of use, and the fact that, for the majority of food items in the FOODfiles, free and added sugars are estimated using objective steps (1–6). This means that detailed knowledge of food composition or production formulation is not required. The use of 24 h dietary recall data from the 2008/09 NZANS (a nationally representative sample) allowed us to generate population-level estimates of free and added sugar intakes, and the large sample size of the survey enabled robust sub-population estimates by sex, age, and ethnicity. 

There are some limitations to the ten-step method. One is that food manufacturers frequently modify product formulations. Consequently the USDA recently announced it will no longer update added and intrinsic sugar values in the USDA nutrient database [23]. The free and added sugar values calculated for FOODfiles data will therefore become out-of-date over time, although this is equally true for many, if not most, nutrients. In addition, the FOODfiles database is regularly updated with new or re-analysed foods. The most recent version in Kai-culator (FOODfiles 2014) included approximately 150 new foods for which free and added sugars needed to be calculated, however this was a relatively minor task. A small proportion (8–11%) of foods required subjective assumptions regarding formulation to be made to estimate free or added sugar content, which compromises the accuracy of the estimates. The problems experienced by the USDA are likely to be less of an issue in New Zealand given our nutrient database contains far fewer food items. Another limitation is that we have not investigated the uncertainty around the free and added sugar estimates derived from subjective assumptions and the potential impact on our population intake estimates. However, approximately 80% of the mean population intake of total sugars and sucrose estimated by the 2008/09 NZANS came from food groups for which free and added sugars were calculated using objective steps for most items within the group (non-alcoholic beverages, sugar and sweets, fruit, dairy, cakes and muffins, vegetables, and biscuits) [11]. Thus, the impact on the accuracy of our intake estimates is likely to be minimal, and almost certainly less than the impact of under-reporting and misreporting [24].

As with all dietary surveys, the self-reported dietary data is subject to recall and reporting bias. While the use of multiple pass methods [18] minimises the risk of reporting bias, the risk of under-reporting of intakes of sugary foods and drinks, particularly by women and obese participants [25], cannot be easily mitigated. Nevertheless the 2008/09 NZANS is the best data available in New Zealand for estimating population nutrient intakes.

## Figures and Tables

**Table 1:** Step allocation for free and added sugar estimates.

Step Number	Number of Foods (%)	
	Free Sugars	Added Sugars
1	685 (24.6)	657 (23.6)
2	1017 (36.6)	1088 (39.2)
3	419 (15.1)	335 (12.1)
4	203 (7.3)	161 (5.8)
5	39 (1.4)	43 (1.5)
6	180 (6.5)	179 (6.4)
7	39 (1.4)	55 (2.0)
8	113 (4.1)	129 (4.6)
9	7 (0.3)	49 (1.8)
10	77 (2.8)	83 (3.0)
Total objective (steps 1–6)	2543 (91.5)	2463 (88.6)
Total subjective (steps 7–10)	236 (8.5)	316 (11.4)
Total	2779 (100)	2779 (100)

**Table 2:** Sugar intakes in New Zealand adults aged 15 years and older in 2008/09 by sugar category, age, and sex (median (25th, 75th percentile)).

Age Group (Years)	All	Male	Female
**Added sugar (g/day)**			
All	48.9 (29.3, 74.7)	58.1 (36.0, 84.8)	41.7 (25.3, 63.2)
15–18	65.9 (38.4, 92.8)	73.3 (47.5, 98.9)	57.4 (34.1, 85.7)
19–30	61.2 (38.7, 97.0)	69.7 (43.1, 108.6)	55.6 (33.4, 83.4)
31–50	49.7 (30.7, 73.9)	62.1 (40.0, 85.9)	40.1 (26.2, 60.9)
51–70	41.0 (24.4, 62.7)	46.0 (29.5, 72.6)	35.7 (21.7, 56.0)
71+	37.2 (24.5, 55.1)	45.9 (32.3, 64.7)	32.2 (20.2, 45.4)
**Free sugar (g/day)**			
All	57.3 (35.0, 84.3)	66.4 (42.7, 94.6)	49.1 (29.7, 73.1)
15–18	76.5 (49.8, 105.6)	85.6 (56.0, 113.3)	68.6 (44.7, 99.2)
19–30	71.7 (47.7, 109.9)	78.1 (56.0, 121.9)	62.4 (41.9, 93.2)
31–50	58.4 (34.8, 85.4)	70.3 (45.1, 96.6)	47.9 (29.2, 69.5)
51–70	49.4 (29.4, 72.9)	54.4 (35.1, 80.2)	43.2 (25.9, 63.0)
71+	43.3 (29.0, 61.3)	51.9 (37.5, 71.4)	36.8 (24.9, 53.5)
**Total sugars ^1^ (% energy)**			
All	21.0 (16.9, 25.4)	19.6 (15.7, 24.0)	22.2 (18.3, 26.4)
15–18	22.0 (16.8, 27.1)	20.2 (14.6, 25.4)	23.9 (19.2, 28.7)
19–30	21.9 (17.7, 26.3)	20.1 (15.9, 24.6)	23.8 (19.7, 27.0)
31–50	20.4 (16.5, 24.5)	19.5 (15.1, 23.6)	21.3 (17.3, 25.6)
51–70	20.4 (16.7, 25.2)	19.5 (15.5, 23.8)	21.7 (18.2, 26.4)
71+	22.1 (18.3, 26.0)	21.1 (17.3, 25.1)	23.3 (19.6, 26.8)
**Sucrose (% energy)**			
All	9.6 (7.0, 12.3)	9.0 (6.6, 11.6)	10.0 (7.4, 12.8)
15–18	10.6 (7.6, 13.9)	9.5 (6.3, 12.8)	11.6 (9.1, 15.0)
19–30	10.3 (8.1, 13.3)	9.4 (7.2, 12.0)	11.1 (9.0, 14.0)
31–50	9.3 (7.0, 12.0)	8.9 (6.8, 11.5)	9.6 (7.2, 12.6)
51–70	8.9 (6.4, 11.9)	8.4 (6.0, 11.4)	9.3 (7.0, 12.0)
71+	9.4 (7.2, 11.7)	9.1 (6.9, 11.5)	9.7 (7.3, 12.0)
**Added sugar (% energy)**			
All	9.6 (6.3, 13.6)	9.6 (6.3, 13.3)	9.6 (6.2, 13.8)
15–18	11.8 (7.8, 16.7)	11.2 (7.5, 15.3)	12.4 (8.2, 18.6)
19–30	11.5 (7.8, 16.0)	10.8 (7.0, 15.7)	12.2 (8.2, 16.2)
31–50	9.4 (6.4, 13.1)	9.5 (6.8, 13.0)	9.1 (6.1, 13.2)
51–70	8.5 (5.2, 12.2)	8.4 (5.3, 12.3)	8.6 (5.2, 12.2)
71+	8.7 (6.0, 11.8)	8.8 (6.6, 12.0)	8.4 (5.6, 11.5)
**Free sugar (% energy)**			
All	11.1 (7.3, 15.4)	11.0 (7.6, 14.9)	11.3 (7.2, 15.8)
15–18	14.2 (9.7, 19.2)	13.0 (8.3, 17.5)	15.2 (10.9, 20.6)
19–30	13.4 (9.8, 18.1)	12.1 (9.6, 17.8)	14.1 (9.9, 18.1)
31–50	10.8 (7.2, 14.8)	10.9 (7.8, 14.3)	10.8 (6.9, 15.2)
51–70	9.8 (6.2, 14.1)	10.1 (5.8, 14.0)	9.7 (6.4, 14.1)
71+	10.0 (7.1, 13.6)	10.1 (7.6, 13.8)	9.9 (6.7, 13.3)

^1^ Total sugars were defined as all naturally occurring and added sugars in food.

**Table 3:** Percentage (95% CI) of New Zealand adults aged 15 years and older in 2008/09 meeting recommendations for free sugar intakes by sex and age group.

Age Group (Years)	All	Male	Female
**Added sugar <10% energy**			
All	52.7 (50.5, 54.8)	53.4 (50.2, 56.6)	52.0 (49.1, 54.9)
15–18	37.1 (32.8, 41.6)	40.8 (34.3, 47.6)	33.3 (30.0, 39.1)
19–30	39.8 (34.6, 45.2)	43.3 (35.7, 51.2)	36.4 (29.7, 43.8)
31–50	54.7 (50.8, 58.5)	54.5 (48.6, 60.2)	54.9 (49.5, 60.1)
51–70	60.7 (56.3, 64.9)	60.8 (54.4, 66.8)	60.6 (54.9, 66.1)
71+	61.3 (57.4, 65.1)	60.4 (54.4, 66.1)	62.0 (57.3, 66.5)
**Free sugar <10% energy**			
All	41.8 (39.7, 44.0)	41.0 (37.9, 44.3)	42.6 (39.9, 45.4)
15–18	27.0 (22.9, 31.5)	32.3 (25.9, 39.4)	21.5 (16.9, 26.9)
19–30	27.2 (22.7, 32.1)	28.5 (22.0, 36.1)	25.9 (20.4, 32.4)
31–50	43.5 (39.6, 47.5)	41.5 (35.8, 47.4)	45.3 (40.1, 50.6)
51–70	51.5 (47.1, 55.6)	49.7 (43.1, 56.2)	53.2 (47.6, 58.8)
71+	50.2 (46.1, 54.3)	48.6 (42.6, 54.6)	51.5 (46.7, 56.3)
**Added sugar <5% energy**			
All	17.2 (15.5, 18.9)	16.9 (14.6, 19.4)	17.4 (15.3, 19.7)
15–18	10.1 (7.5, 13.6)	11.4 (7.2, 17.6)	8.8 (6.1, 12.6)
19–30	13.4 (10.0, 17.7)	14.9 (10.0, 21.7)	12.0 (8.0, 17.7)
31–50	16.3 (13.6, 19.5)	15.3 (11.7, 19.9)	17.3 (13.5, 21.8)
51–70	23.2 (19.6, 27.3)	23.3 (18.1, 29.5)	23.2 (18.7, 28.3)
71+	16.4 (13.8, 19.5)	12.6 (9.7, 16.3)	19.5 (15.2, 24.7)
**Free sugar <5% energy**			
All	11.9 (10.5, 13.4)	12.1 (10.1, 14.5)	11.7 (10.0, 13.6)
15–18	6.6 (4.3, 9.9)	8.5 (4.7, 14.8)	4.6 (2.8, 7.6)
19–30	7.6 (5.1, 11.2)	8.3 (4.9, 13.8)	7.0 (4.1, 11.8)
31–50	12.8 (10.3, 15.7)	12.4 (9.0, 16.7)	13.1 (9.9, 17.1)
51–70	15.6 (12.7, 19.1)	17.0 (12.3, 23.0)	14.3 (10.9, 18.7)
71+	11.3 (8.9, 14.2)	8.6 (6.3, 11.8)	13.4 (9.5, 18.6)

## Appendix A. Detailed 10-Step Method for Estimating Free Sugars

Step 1 All foods with a total sugar content of 0 g were assigned 0 g free sugars.
Step 2 All foods in the following groups were assigned 0 g of free sugars:
  (a) All spices and herbs.
  (b) All fats and oils.
  (c) All plain cereal grains, pasta, oats, rice, and flours.
  (d) All plain breads, including pizza bases, pita, naan, and English muffins (excluding gluten free breads).
  (e) Unfilled plain pastries which do not contain, chocolate, nuts, or dried fruits.
  (f) Eggs and egg products (except egg-based desserts).
  (g) Fresh fruit; unsweetened dried fruit; fruit canned in syrup, sweetened with artificial sweetener only; and fresh vegetables.
  (h) Nuts and seeds (excluding nut/seed bars and coated nuts/seeds).
  (i) Fresh meat, fresh seafood, tofu, and unsweetened legumes (fresh, dried, or processed).
  (j) Non-sweetened coffees, tea, and alcohol (excluding liqueurs and mixers).
  (k) Non-sugar-sweetened milk and dairy products (including those sweetened with artificial sweeteners only).
Step 3 All foods in the following groups had 100% of sugar assigned to free sugars:
  (a) All sugars and syrups.
  (b) All confectionary (excluding chocolate) and potato chips.
  (c) Coffee and beverage bases.
  (d) All fruit juices, purees, concentrates, and jams (both sweetened and unsweetened varieties), including tomato pastes, sauces, and purees.
  (e) Sugar-sweetened soft drinks, sports drinks, flavoured waters, and energy drinks.
  (f) Non-cream based liqueurs.
  (g) All baked goods such as biscuits, cakes, buns, and crackers that did not contain fruit, chocolate, or dairy products.
  (h) Gluten-free breads.
  (i) All breakfast cereals and cereal bars which do not contain fruit pieces, chocolate, or dairy products.
  (j) Stock powder—dry or made up with water.
  (k) Sauces and dressings, excluding pasta sauces and those that are vegetable-based such as pickles.
  (l) Processed meats including, pies, pastries, crumbed/battered meat, AND seafood.
  (m) Soy beverages and soy yoghurt without added fruits.
Step 4a Calculation based on standard recipe used in the food composition database where free sugar contents of ALL ingredients were available from steps 1 to 3.

or

Step 4b Calculation based on known proportion of canned fruits and their juices/syrups.
Step 4a) Free sugars per 100 g (FS_100 g_) is given by the following formula:
  $$FS_{100g}=\frac{\sum_{i=1}^{j}W_{i}\times FS_{i}}{\sum_{i=1}^{j}(100\%+\%W_{\Delta })}$$
  where *W_i_* equates to the weight of the *i*th ingredient of the recipe, *FS_i_* is the quantity of free sugars in the *i*th ingredient of the recipe, and % *W*∆ is the percentage weight change of the recipe from cooking.
Step 4b) FS_100 g_ of undrained canned fruits were determined using the following formula:
  $$FS_{100g=\;}S_{T\;}-\left(S_{R}\times \;{\%}_{R}\right)$$
  where *S_T_* is the total sugar, *S_R_* is the sugar content of the raw fruit, and %*_R_* is the proportion of raw fruit in the can of fruit.
Step 5 Calculation based on comparison with values from the unsweetened variety. Free sugars per 100 g (*FS***_100 g_**) is given by the following formula:
  $$FS_{100g}=\frac{100\;\times \left(S_{US}\right)-\left(S_{T}\right)}{S_{US}-100}$$
  where *S_US_* is the total sugar content per 100 g of the unsweetened variety of the food, and *S_T_* is the total sugar for the food item that free sugars is to be estimated for.
Step 6 Calculate free sugars from mono- and disaccharide content. Free sugars were calculated as total sugar minus lactose. Lactose was subtracted from total sugars for all foods with the exception of potato chips, other chip varieties, and confectionary (excluding chocolate, fudge, and toffee). In this instance lactose was determined to be a sweetener rather than an intrinsic sugar, since foods such as these are considered to be discretionary.
Step 7 Free sugars estimated using borrowed values from similar foods that had previously been determined using steps 1–6 OR using an overseas database. Free sugars were estimated by determining the proportion of free sugars to total sugar from the borrowed food, then using this proportion on the food to be estimated with the equation: ‘total sugars *×* %FS (from the borrowed food)’.
Step 8 Subjective estimation based on the best available information regarding ingredients and/or common recipes and/or assumptions. *See* *Table A1* *for list of additional assumptions.*
Step 9 Free sugar estimation based on standard recipe used in the food composition database where free sugar content of ANY of the ingredients was determined by using steps 5–10 (i.e., step 4 was repeated).
Step 10 50% of total sugars assumed to be free sugars. Step 10 was used when it was not possible to determine free sugars using steps 1–9. This step was predominantly used for takeaways, such as pizzas and burgers. Additionally, it was used on some soups, sauces, and miscellaneous foods.

**Table A1:** Additional Assumptions.

Food Category	Sub Category	Assumptions	Step	Rule	Example
Canned Fruit	Undrained (syrup or juices)	Free sugar intake of canned fruit was estimated by working out the average proportion of fruit per can (looking at various brands, i.e., Watties, Oak, Golden Circle, Pams, Budget, and Dole). Proportion was multiplied by the amount of sugar found in the whole fruit and then this total amount was taken away from the total sugars. The rest of the sugar was assumed to come from fruit juice or other free sugars.	4b		L205 Pineapple canned in Light Syrup L205 contains 16.3 g of total sugar. A raw pineapple (L144) contains 11.4 g of total sugar. Pineapple in syrup was found to have an average of 65% fruit on average, across all brands. Therefore; FS = 16.3 − (11.4 × 0.65) = 8.91 g
	Drained (syrup)	Compared with raw version of the fruit.	5		
	Drained (Juice)	Assume that after draining the juice that there are zero free sugars.	2		
Soup	With recipes	Free sugar determined through recipe proportions.	4		
	Containing only lactose	Soups only containing sugar from lactose assumed to have zero free sugars.	6	Total sugar − lactose	V1005 Soup, seafood chowder, ready to serve, pouch. Contains 1.5 g of total sugar and 1.5 g of lactose 1.5 − 1.5 = 0 g Free sugar
	Commercial soups	Commercials soups that were comparable to homemade varieties were determined via the proportion of free sugars in the homemade variety.	7		Free sugar content of commercial pumpkin soups e.g., V1001 assumed to be 7% based on homemade pumpkin soup V34
	Tomato soups	Based on ingredients list of a number of tomato soups, which stated approximately 90% tomatoes in ingredients.	8	10% free sugar	
	Condensed tomato Soups	Based on ‘regular’ tomato soups, proportion of free sugars was increased based on the increased concentration of ingredients.	8	20% free sugar	
	‘Other soups’	Soups not covered in above categories assumed to be 50% free sugars.	10	50% free sugar	
Yoghurt	With puree	Such as ‘fruit corners’, assumed to be all free sugar minus lactose	6	100% free sugar	
	‘Other’	Flavoured with fruit pieces—assumed to be apricot (stewed without sugar) unless otherwise stated due to apricot flavour’s popularity and similarity (sugar content) to other flavours such as mango or peach. Fruit proportion was based on yoghurts currently available for purchase.	8	7% Fruit	F55 Yoghurt, assorted fruits & flvrflavour, regular fat, sweetened. Total sugars = 14.1/100g. Lactose = 3.9/100 g Assumed 7% stewed apricot (L25 with 5.4/100 g of sugar) =14.1 − (5.4 × 0.07) − 3.9 =9.82/100 g free sugar
Muesli bars	With fruit pieces OR fruit and nut bars	Assumed to have 20% dried fruit based on current muesli bars available for purchase. Total sugars from dried apricots (L26, 44.9/100 g total sugar) were used if the fruit was not specified.	8	20% dried fruit	U1 Muesli bar, apricot Total sugar = 15.51 Lactose = 0.4 =15.51 − (0.20 × 44.9) − 0.4 =6.13/100 g free sugars
	Fruit-filled	Treated as a puree so assumed to be all free sugars (minus any lactose).	3	100% free sugar	
Currant breads	All	Assumed to have zero added sugars as once a standardised % of dried fruit was removed (20%), the remaining sugar level was comparable to that of normal bread.	8	0% free sugars	
Discretionary foods		Confectionary (excluding chocolate) such potato chips etc. were considered to be 100% free sugars, even if derived from food ingredients such as milk.	3	100% free sugars	
	Chocolate	Milk solids were assumed to be an inherent part of the product, i.e., not being used as a sweetener. Therefore, free sugar content of plain chocolate was total sugar − lactose. Any fruit or nut varieties were determined via Step 8 through proportioning.	6	Total sugar − lactose for plain chocolate	
Flavoured drink powders	E.g., Milo, Ovaltine	Considered a drink sweetener, therefore 100% free sugars (including lactose).	3	100% free sugars	
Breakfast cereals	All cereals	Determined by ingredients listed and their proportions. Cereals no longer available were matched to a similar, currently available, related product. All dried fruits (excluding dried purees) which were not available on FOODfiles were matched to one of four dried fruits (sultanas, raisins, dried apricots, and cranberries) Dried purees were assumed to be 100% free sugars. If proportions of fruit were not broken down into each individual food type, then it was assumed to have equal proportions of each fruit, e.g., an ingredient list stating ‘dried fruit 20% (dried apricots and sultanas)’ would be assumed to have 10% of both dried apricots and sultanas.	8		D1019 Muesli, ‘Natural fruit & five grains’, Sanitarium 20.5/100 g of total sugar 0 g of lactose Listed as having 22% dried apriots and sultanas however proportions of each are not listed. Assumed 11% dried apricots (L26 with 44.9/100 g sugars) Assumed 11% sultanas (L173 with 73.2 g/100 g sugars) =20.5 − (44.9 × 0.11) − (73.2 × 0.11) = 7.5/100 g free sugar
