# Erratum: Estimating Free and Added Sugar Intakes in New Zealand; *Nutrients* 2017, *9*, 1292

**DOI:** 10.3390/nu10050638

**Published:** 2018-05-18

**Authors:** Rachael Kibblewhite, Alice Nettleton, Rachael McLean, Jillian Haszard, Elizabeth Fleming, Devonia Kruimer, Lisa Te Morenga

**Affiliations:** Department of Human Nutrition, University of Otago, P.O. Box 56, Dunedin 9054, New Zealand; kibra436@student.otago.ac.nz (R.K.); netal083@student.otago.ac.nz (A.N.); Rachael.mclean@otago.ac.nz (R.M.); jill.haszard@otago.ac.nz (J.H.); liz.fleming@otago.ac.nz (E.F.); devonia.kruimer@otago.ac.nz (D.K.)

The authors have requested that the following changes be made to their paper [1].

In Figure 1, the caption was changed to “Figure 1. 10–step method for estimating free sugars content (adapted from Louie et al. 2015 [14])” [2].

In Appendix A, “This appendix details how we used and adapted the 10-step methodology for estimating added sugars described by Louie et al. 2015 [14] to calculate free sugars in the New Zealand food composition database, based on analytical data on total sugars and ingredients in food products. We used the unmodified Louie method to estimate added sugars in the New Zealand food composition database as reported in this paper” [2] was inserted in front of the Appendix A title. Further, “adapted from Louie et al., 2015 [14]” [2] was added after the title.

“as per Step 1 of Louie et al., 2015 [14]” [2] was added in Step 1.

“as per Step 2 of Louie et al., 2015 [14]” [2] was added in Step 2.

“adapted from Step 3 of Louie et al., 2015 [14]” [2] was added in Step 3.

“as per Step 4 of Louie et al., 2015 [14]” [2] was added in Step 4a; “adapted from Step 4 of Louie et al.2015 [14]” [2] was added in Step 4b.

“as per Step 5 of Louie et al., 2015 [14]” [2] was added in Step 5.

“as per Step 6 of Louie et al., 2015 [14]” [2] was added in Step 6.

“adapted from Step 7 of Louie et al., 2015 [14]” [2] was added in Step 7.

“as per from Step 9 of Louie et al., 2015 [14]” [2] was added as the last sentence of Step 9.

“adapted from Step 10 of Louie et al., 2015 [14]” [2] was added as the last sentence of Step 10.

## Notes

The updated version of Appendix A should therefore read as follows:

## Appendix A

This appendix details how we used and adapted the 10-step methodology for estimating added sugars described by Louie et al. 2015 [14] to calculate free sugars in the New Zealand food composition database based on analytical data on total sugars and ingredients in food products. We used the unmodified Louie method to estimate added sugars in the New Zealand food composition database as reported in this paper.

## Detailed 10-Step Method for Estimating Free Sugars

Step 1 All foods with a total sugar content of 0 g were assigned 0 g free sugars (as per Step 1 of Louie et al. 2015 [14]).
Step 2 All foods in the following groups were assigned 0 g of free sugars: (as per Step 2 of Louie et al. 2015 [14]).
(a) All spices and herbs.
(b) All fats and oils.
(c) All plain cereal grains, pasta, oats, rice, and flours.
(d) All plain breads, including pizza bases, pita, naan, and English muffins (excluding gluten free breads).
(e) Unfilled plain pastries which do not contain, chocolate, nuts, or dried fruits.
(f) Eggs and egg products (except egg-based desserts).
(g) Fresh fruit; unsweetened dried fruit; fruit canned in syrup, sweetened with artificial sweetener only; and fresh vegetables.
(h) Nuts and seeds (excluding nut/seed bars and coated nuts/seeds).
(i) Fresh meat, fresh seafood, tofu, and unsweetened legumes (fresh, dried, or processed).
(j) Non-sweetened coffees, tea, and alcohol (excluding liqueurs and mixers).
(k) Non-sugar-sweetened milk and dairy products (including those sweetened with artificial sweeteners only).
Step 3 All foods in the following groups had 100% of sugar assigned to free sugars: (adapted from Step 3 of Louie et al. 2015 [14]).
(a) All sugars and syrups.
(b) All confectionary (excluding chocolate) and potato chips.
(c) Coffee and beverage bases.
(d) All fruit juices, purees, concentrates, and jams (both sweetened and unsweetened varieties), including tomato pastes, sauces, and purees.
(e) Sugar-sweetened soft drinks, sports drinks, flavoured waters, and energy drinks.
(f) Non-cream based liqueurs.
(g) All baked goods such as biscuits, cakes, buns, and crackers that did not contain fruit, chocolate, or dairy products.
(h) Gluten-free breads.
(i) All breakfast cereals and cereal bars which do not contain fruit pieces, chocolate, or dairy products.
(j) Stock powder—dry or made up with water.
(k) Sauces and dressings, excluding pasta sauces and those that are vegetable-based such as pickles.
(l) Processed meats including, pies, pastries, crumbed/battered meat, AND seafood.
(m) Soy beverages and soy yoghurt without added fruits.
Step 4a Calculation based on standard recipe used in the food composition database where free sugar contents of ALL ingredients were available from steps 1 to 3 (as per Step 4 of Louie et al. 2015 [14]).

or

Step 4b Calculation based on known proportion of canned fruits and their juices/syrups (adapted from Step 4 of Louie et al. 2015 [14]).
Step 4(a) Free sugars per 100 g (FS_100 g_) is given by the following formula:
  $$FS_{100g}=\frac{\sum_{i=1}^{j}W_{i}\times FS_{i}}{\sum_{i=1}^{j}(100\%+\%W_{\Delta })}$$
  where *W_i_* equates to the weight of the *i*th ingredient of the recipe, *FS_i_* is the quantity of free sugars in the *i*th ingredient of the recipe, and % *W*∆ is the percentage weight change of the recipe from cooking.
Step 4(b) FS_100 g_ of undrained canned fruits were determined using the following formula:
  $$FS_{100g}=S_{T}-\left(S_{R}\times {\%}_{R}\right)$$
  where *S_T_* is the total sugar, *S_R_* is the sugar content of the raw fruit, and %*_R_* is the proportion of raw fruit in the can of fruit.
Step 5 Calculation based on comparison with values from the unsweetened variety. Free sugars per 100 g (*FS*_100 g_) is given by the following formula:
  $$FS_{100g}=\frac{100\times \left(S_{US}\right)-\left(S_{T}\right)}{S_{US}-100}$$
  where *S_US_* is the total sugar content per 100 g of the unsweetened variety of the food, and *S_T_* is the total sugar for the food item that free sugars is to be estimated for (as per Step 5 of Louie et al. 2015 [14]).
Step 6 Calculate free sugars from mono- and disaccharide content. Free sugars were calculated as total sugar minus lactose. Lactose was subtracted from total sugars for all foods with the exception of potato chips, other chip varieties, and confectionary (excluding chocolate, fudge, and toffee). In this instance lactose was determined to be a sweetener rather than an intrinsic sugar, since foods such as these are considered to be discretionary (as per Step 6 of Louie et al. 2015 [14]).
Step 7 Free sugars estimated using borrowed values from similar foods that had previously been determined using steps 1–6 OR using an overseas database. Free sugars were estimated by determining the proportion of free sugars to total sugar from the borrowed food, then using this proportion on the food to be estimated with the equation: ‘total sugars *×* %FS (from the borrowed food)’ (adapted from Step 7 of Louie et al. 2015 [14]).
Step 8 Subjective estimation based on the best available information regarding ingredients and/or common recipes and/or assumptions. *See Table A1 for list of additional assumptions*.
Step 9 Free sugar estimation based on standard recipe used in the food composition database where free sugar content of ANY of the ingredients was determined by using steps 5–10 (i.e., step 4 was repeated) (as per from Step 9 of Louie et al. 2015 [14]).
Step 10 50% of total sugars assumed to be free sugars. Step 10 was used when it was not possible to determine free sugars using steps 1–9. This step was predominantly used for takeaways, such as pizzas and burgers. Additionally, it was used on some soups, sauces, and miscellaneous foods (adapted from Step 10 of Louie et al. 2015 [14]).

The authors apologize for any inconvenience caused to the readers by the changes, stating it does not affect the scientific results. The original manuscript will remain online on the article webpage, with a reference to this Erratum.

## References

[1] Kibblewhite R., Nettleton A., McLean R., Haszard J., Fleming E., Kruimer D., Te Morenga L. (2017). Estimating free and added sugar intakes in New Zealand. Nutrients.

[2] Louie J.C.Y., Moshtaghian H., Boylan S., Flood V.M., Rangan A., Barclay A., Brand-Miller J., Gill T. (2015). A systematic methodology to estimate added sugar content of foods. Eur. J. Clin. Nutr..

